# Meningitis, meningoencephalitis and encephalitis in Bern: an observational study of 258 patients

**DOI:** 10.1186/s12883-021-02502-3

**Published:** 2021-12-06

**Authors:** Anamaria Ungureanu, Julia van der Meer, Antonela Bicvic, Lena Abbuehl, Gabriele Chiffi, Léonore Jaques, Franziska Suter-Riniker, Stephen L. Leib, Claudio L. A. Bassetti, Anelia Dietmann

**Affiliations:** 1 Department of Neurology, University Hopsital and University of Bern, Inselspital, Bern, Switzerland; 2 Institute for Infectious Disease, University of Bern, Bern, Switzerland

**Keywords:** Encephalitis, Meningoencephalitis, Meningitis, Sleep-wake disturbances, Tick-borne encephalitis

## Abstract

**Background:**

Depending on geographic location, causes of encephalitis, meningoencephalitis and meningitis vary substantially. We aimed to identify the most frequent causes, clinical presentation and long-term outcome of encephalitis, meningoencephalitis and meningitis cases treated in the Inselspital University Hospital Bern, Switzerland.

**Methods:**

In this monocentric, observational study, we performed a retrospective review of clinical patient records for all patients treated within a 3-year period. Patients were contacted for a telephone follow-up interview and to fill out questionnaires, especially related to disturbances of sleep and wakefulness.

**Results:**

We included 258 patients with the following conditions: encephalitis (18%), nonbacterial meningoencephalitis (42%), nonbacterial meningitis (27%) and bacterial meningoencephalitis/meningitis (13%). Herpes simplex virus (HSV) was the most common cause of encephalitis (18%); tick-borne encephalitis virus (TBEV) was the most common cause of nonbacterial meningoencephalitis (46%), enterovirus was the most common cause of nonbacterial meningitis (21%) and *Streptococcus pneumoniae* was the most common cause of bacterial meningoencephalitis/meningitis (49%). Overall, 35% patients remained without a known cause. After a median time of 16 months, 162 patients participated in the follow-up interview; 56% reported suffering from neurological long-term sequelae such as fatigue and/or excessive daytime sleepiness (34%), cognitive impairment and memory deficits (22%), headache (14%) and epileptic seizures (11%).

**Conclusions:**

In the Bern region, Switzerland, TBEV was the overall most frequently detected infectious cause, with a clinical manifestation of meningoencephalitis in the majority of cases. Long-term neurological sequelae, most importantly cognitive impairment, fatigue and headache, were frequently self-reported not only in encephalitis and meningoencephalitis survivors but also in viral meningitis survivors up to 40 months after acute infection.

**Supplementary Information:**

The online version contains supplementary material available at 10.1186/s12883-021-02502-3.

## Background

Encephalitis/meningoencephalitis is an inflammation of the brain parenchyma with or without involvement of the meningeal structures. Meningitis is either a severe acute bacterial infection or less fulminant of viral origin [1, 2]. Encephalitis is a serious and sometimes life-threatening disease that is often associated with long-term morbidity [3–5]. Considering significant geographic variation, encephalitis has a worldwide incidence between 1 and 13 cases/100.000/year [6, 7]. In Switzerland, tick-borne encephalitis virus (TBEV) is one of the most frequent causes of meningoencephalitis [8], whereas in the United Kingdom, herpes simplex virus (HSV) is the most common infectious cause of encephalitis [3]. Other cases of encephalitis are caused by infectious agents, including varicella zoster virus (VZV) and *Mycobacterium tuberculosis,* or by cellular or humoral autoimmune processes [3]. In 37-67% of patients, the cause of encephalitis remains unknown [3–5, 9]. Similarly, in a large observational cohort study from the United Kingdom of nonbacterial meningitis cases, 42% remained without known cause, whereas enterovirus was the most common pathogen [1].

Infectious encephalitis and meningoencephalitis are associated with a high incidence of severe and debilitating long-term sequelae, whereas outcomes after autoimmune encephalitis are variable [10–14]. In contrast, viral meningitis is considered a benign, self-limiting illness; however, increasing evidence suggests that this may often not be the case [1]. In particular, signs and symptoms such as fatigue, excessive daytime sleepiness (EDS) or disturbed nighttime sleep/insomnia are frequently reported in routine clinical follow-up consultations. Fatigue as a long-term sequelae, without a detailed definition, has been described in the literature [15, 16] However, studies evaluating these important clinical issues have been published only recently [17, 18].

The aims of our study were to (1) determine the most common causes of encephalitis, meningoencephalitis and meningitis in our hospital and to (2) investigate the frequency of long-term sequelae with a focus on disorders of sleep and wakefulness (SWD).

## Methods

The study was designed as a monocentric, observational study and contained two parts. The first part comprised a retrospective analysis of medical records from all patients diagnosed with any acute encephalitis, meningoencephalitis or meningitis treated in the Inselspital in Bern, Switzerland, a tertiary care university hospital with a population base of 1.5 million inhabitants. Ethical approval was given by the local Ethics Committee (Kantonale Ethikkommission Bern ID 2018-01523). Research governance approval was given at the University Hospital Inselspital, Bern, Switzerland. Medical records were only used if written general consent for research projects was available or if patients gave oral and written informed consent in the course of the telephone interview. In the second part, patients were contacted for a telephone follow-up interview and were asked to fill out and return questionnaires by mail. Oral informed consent was given during telephone interviews, and written informed consent was returned by mail.

### Study database

Study data were collected and managed using REDCap electronic data capture tools hosted at the Department of Neurology, University Hospital and University of Bern, Inselspital, Bern, Switzerland [19, 20]. REDCap (Research Electronic Data Capture) is a secure, web-based software platform designed to support data capture for research studies, providing 1) an intuitive interface for validated data capture; 2) audit trails for tracking data manipulation and export procedures; 3) automated export procedures for seamless data downloads to common statistical packages; and 4) procedures for data integration and interoperability with external sources.

### Participants and study procedures

Possible and consecutive participants were retrospectively identified from 1.1.2016 until 31.10.2018 by screening the medical record database of the Inselspital University Hospital for ICD-10 Codes A83, A84, A85, B00.4, B01.0, B02.0, B05.0, B26.2, B58.2, G04, and G05 referring to all possible causes of encephalitis, meningoencephalitis or meningitis. Patients were either treated at the Department of Internal Medicine, Department of Neurology and/or the Intensive Care Unit.

Patients were eligible if aged 16 and older and had a diagnosis of encephalitis, meningoencephalitis or meningitis in their medical record, confirmed by lumbar puncture with signs of acute inflammation and/or appropriate pathogen identified either on cerebrospinal fluid (CSF) or blood PCR, serology or culture. The exclusion criteria were primary central nervous system (CNS) vasculitis, cerebral venous thrombosis, brain or spinal cord abscess, active CNS tumour or CNS lymphoma, toxic or metabolic encephalopathy or spongiform encephalopathy.

Diagnosis was reviewed and, if necessary, revised by two neurologists (A.D. and A.U.) according to published definitions [1, 21–23]. In detail, patients with acute onset of headache, fever and/or meningism, CSF leucocyte count > 4 × 10^6^ cells/L and, if possible, detection of an appropriate pathogen by either CSF PCR, blood/CSF serology or blood/CSF culture or throat or rectal swab were classified as having meningitis [1]. Meningoencephalitis was classified when signs of meningitis were present plus altered consciousness and/or focal neurological symptoms and/or abnormal findings in EEG [10]. Encephalitis was defined as altered consciousness for > 24 h with no other cause and evidence of CNS inflammation, demonstrated by at least two of the following criteria: fever, seizures or focal neurological findings attributable to the brain parenchyma, CSF pleocytosis (CSF leucocyte count > 4 × 10^6^ cells per L), EEG findings suggestive of encephalitis and/or neuroimaging findings suggestive of encephalitis [21]. For TBE, the following case definition was applied: patients with symptoms of CNS inflammation (meningitis, meningoencephalitis or encephalitis criteria), history of possible exposure or tick bites and detection of TBE-specific IgM and IgG antibodies in serum using the SERION ELISA classic FSME virus/TBE virus IgG and IgM kit (Virion\Serion, Würzburg, Germany). In cases where only IgM antibodies are detected, a follow-up sample is needed to demonstrate IgG seroconversion and thereby establish the diagnosis [22, 23].

Detection of TBE viral nucleic acid in blood by PCR or isolation of TBE virus was not performed.

The FilmArray® ME Panel (BioFire, bioMerieux, Salt Lake City, USA) was used. Two hundred microlitres of CSF was subjected to FilmArray® ME Panel testing according to the manufacturer’s instructions. The FilmArray® ME Panel test consists of automated sample homogenization and nucleic acid extraction, reverse transcription, and nucleic acid amplification. The FilmArray® ME Panel identifies 14 common agents of community-acquired ME: *Escherichia coli K1, Haemophilus influenzae*, *Listeria monocytogenes*, *Neisseria meningitidis, Streptococcus pneumoniae*, *Streptococcus agalactiae*, cytomegalovirus (CMV), enterovirus (EV), herpes simplex virus type 1 (HSV-1), herpes simplex virus type 2 (HSV-2), human herpesvirus type 6 (HHV-6), human parechovirus (HPeV), varicella zoster virus (VZV), and *Cryptococcus neoformans*/gattii from CSF.

Bacterial meningitis cases were diagnosed by culture.

*Borrelia Burgdorferi*: serological testing of IgG and IgM using IgG and IgM recomWell ELISA (recomWell, Mikrogen) according to the manufacturer’s instructions. In the case of positive screening test results, the CSF/serum antibody index was determined using IgG and IgM ELISA Virion/Serion (Würzburg, Germany) according to the manufacturer’s instructions. In this case, the presence of Borrelia-specific antibodies in simultaneously sampled serum and CSF probes was determined.

*Treponema pallidum* infection: reactive VRDL in CSF and/or a positive CSF intrathecal T pallidum antibody index.

Preexisting diseases, immune suppressive state and signs and symptoms pre- and in the hospital were taken from the medical records. Furthermore, findings in the neurological examinations, laboratory findings and microbiological results were taken from the clinical record. At hospital discharge, a modified Rankin score (mRS) was taken from the clinical record if available or calculated as described in the clinical record.

### Follow-up

Clinical outcomes and subjective long-term sequelae were assessed with a standardized interview via telephone (A.U., L.J.) from October 2019 until February 2020. Patients were contacted twice, first for information and informed consent procedures and a second time for the interview. Following questionnaires were sent out immediately after the telephone interview to the study participants evaluating sleep-wake-disorders including Epworth Sleepiness Scale (ESS, self-administered evaluation of daytime sleepiness )[24], Fatigue Severity Scale (FSS, self-administered evaluation of fatigue, designed to differentiate fatigue from clinical depression )[25], Insomnia Severity Index (ISI, self-administered evaluation of severity of both nighttime and daytime components of insomnia )[26], Beck Depression Inventory II (BDI II, self-administered evaluation of severity of depression )[27]. Cut-offs for pathological scores were set according to the literature: ESS > 9, FSS > 4, ISI > 7, BDI II > 8. All questionnaires are frequently used in clinical routine as well as for scientific purposes and have been frequently used in or validated for Swiss patients [25, 28–31]. Furthermore, a selected set of individual questions regarding SWD from the Bern Sleep Questionnaire were sent to the study participants (see supplementary data). The full questionnaire used for clinical routine in our sleep centre has been described elsewhere [31]. Four questions regarding restless leg syndrome (RLS) were included according to diagnostic consensus criteria [32].

### Statistical analysis

Nonparametric continuous data were analysed by using Kruskal–Wallis tests. Categorical data were analysed by using χ^2^ test. Correlation coefficients (R values) were obtained using Pearson’s correlation analysis. A *p* value of less than 0.05 was considered statistically significant. Missing data were not imputed. We statistically analysed data using Stata/MP 16.0 and R 3.6.1, and graphs were drawn by GraphPad Prism 8, Stata/MP 16.0 and R 3.6.1.

## Results

**Fig. 1:**
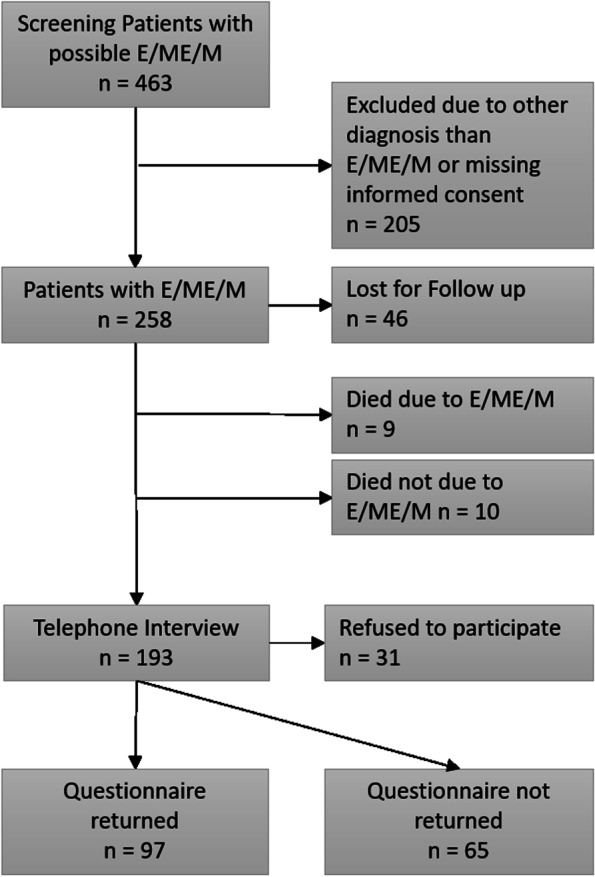
Flow chart of patient recruitment and data acquisition. Legend: *E/ME/M* encephalitis, meningoencephalitis or meningitis

From a total of 463 screened patient medical records, 258 patients were included in the retrospective analysis of the study (Fig. 1).

### Distribution of diagnoses and causes

In total, as shown in Table 1, during the 34-month period, 258 patients were treated for encephalitis (46, 18%), nonbacterial meningoencephalitis (109, 42%), nonbacterial meningitis (70, 27%) or bacterial meningoencephalitis/meningitis (33, 13%).

**Table 1:** Demographics, clinical signs and symptoms and summary of cerebrospinal fluid investigations of all study patients and comparison between groups of encephalitis, nonbacterial meningoencephalitis, nonbacterial meningitis and bacterial meningoencephalitis or meningitis defined by the clinical syndrome

	All	Encephalitis	Nonbacterial Meningo- encephalitis	Nonbacterial Meningitis	Bacterial Meningoencephalitis or Meningitis	*p* value^b^
Total *n* (%)	258 (100)	46 (17.8)	109 (42.2)	70 (27.1)	33 (12.8)	
Sex female *n* (%)	126 (48.8)	18 (39.1)	53 (48.6)	41 (58.6)	14 (42.4)	0.18
Median age (IQR)	51.5 (33)	60 (24)	56 (36)	38 (28)	53 (19)	< 0.0001
Median days from clinical onset to hospital admission (IQR)	5 (8)	5.5 (18)	7 (10)	4 (5)	2 (5)	< 0.0003
Symptoms or clinical signs						
Headache^a^	183 (71, 65-76)	12 (26, 15-41)	85 (78, 69-85)	63 (90, 80-95)	23 (70, 52-83)	
Fever	150 (58, 52-64)	17 (37, 24-52)	73 (67, 58-75)	38 (54, 42-66)	22 (67, 49-81)	
Meningism	77 (30, 25-36)	2 (4, 1-16)	29 (27, 19-36)	27 (39, 28-50)	19 (58, 40-73)	
Photo−/Phonophobia	47 (18, 14-23)	0 (0)	16 (15, 9-23)	27 (39, 9-23)	4 (12, 5-28)	
Nausea/vomiting	98 (38, 32-44)	8 (17, 9-31)	40 (37, 28-46)	40 (57, 45-68)	10 (30, 17-48)	
Epileptic Seizures	73 (29, 23-34)	27 (59, 44-72)	32 (29, 22-39)	2 (2, 1-11)	12 (36, 22-54)	
Sensory/motor deficits	66 (26, 21-31)	12 (26, 15-41)	38 (35, 27-44)	7 (10, 5-20)	9 (27, 15-45)	
Confusion	74 (29, 24-35)	27 (59, 44-72)	31 (28, 21-38)	6 (9, 4-18)	10 (30, 17-48)	
Cognitive impairment	70 (27, 22-33)	24 (52, 38-66)	36 (33, 25-42)	4 (6, 2-14)	6 (18, 8-35)	
Abnormal behaviour	53 (21, 16-26)	23 (50, 36-64)	23 (21, 14-30)	3 (4, 1-13)	4 (12, 5-28)	
Aphasia	55 (21, 17-27)	16 (35, 23-50)	35 (32, 24-42)	0 (0)	4 (12, 5-28)	
Vertigo	48 (19, 14-24)	9 (19, 11-34)	28 (26, 18-35)	9 (13, 7-23)	2 (6, 2-21)	
Gait disturbance	45 (17, 13-23)	11 (24, 14-38)	28 (26, 18-35)	2 (3, 1-11)	4 (12, 5-28)	
Cerebellar signs	36 (14, 10-19)	10 (22, 12-36)	24 (22, 15-31)	1 (1, 0-10)	1 (3, 1-19)	
Cranial nerve dysfunction	37 (14, 11-19)	2 (4, 1-16)	24 (22, 15-31)	4 (6, 2-14)	7 (21, 10-38)	
Impaired consciousness	101 (39, 33-45)	27 (59, 44-72)	46 (42, 33-52)	2 (3, 1-11)	26 (79, 62-90)	
GCS < 15	91 (35, 30-41)	23 (54, 40-68)	34 (37, 28-46)	1 (0-10)	24 (76, 58-87)	
GCS median (IQR)	9 (6)	8 (9)	10 (6)	13 (0)	9 (3)	
Mechanical ventilation	45 (17, 13-23)	12 (26, 15-41)	17 (16, 10-24)	0 (0)	16 (49, 32-65)	
mRS (IQR)	2 (2)	3 (2)	2 (2)	1 (1)	3 (1)	
CSF results						
Median leucocyte count (×10^6^ per L, IQR)	81.5 (190.5)	14 (55)	75.5 (148)	96 (194)	1218.5 (5946)	< 0.0001
Proportion lymphocytes of white blood cells (%, IQR)	92 (49.5)	99 (7)	94 (32)	91.5 (25)	10 (23)	< 0.0001
Median protein (g/L, IQR)	0.78 (0.61)	0.66 (0.54)	0.79 (0.43)	0.58 (0.41)	3.36 (6.63)	< 0.0001
Median lactate (mmol/L, IQR)	2.5 (1.09)	2.2 (0.8)	2.5 (0.75)	2.3 (1)	13 (11.9)	< 0.0001

*Legend*: ^a^Data are the number of patients with each sign or symptom on admission and/or course of acute disease (%, 95 confidence interval CI), *IQR* interquartile range 75-25, ^b^Kruskal–Wallis equality-of-populations rank test comparing all 4 diagnostic groups. Categorical data were analysed by χ^2^ test comparing all 4 diagnostic groups. *CSF* cerebrospinal fluid, *GCS* Glasgow Coma Scale, *mRS* modified Rankin Scale

As shown in Table 2, the most frequent causes overall were of infectious origin (57%); in 5%, an immune-mediated cause was found, and 36% remained without a known cause.

**Table 2:** Confirmed causes of encephalitis, meningoencephalitis or meningitis

Total *n* (%)	All	Encephalitis	Meningoencephalitis	Meningitis
	258 (100)	46 (17.8)	127 (49.2)	85 (32.9)
*Infectious cause*	148 (57.4)	15 (32.6)	89 (70.1)	45 (52.9)
** Viral**	**114 (77)**	**14 (93.3)**	**71 (79.8)**	**30 (68.2)**
Tick-borne Encephalitis Virus	65 (57)	1 (7.1)	58 (81.7)	6 (20.7)
Enterovirus	21 (18.4)	1 (7.1)	3 (4.2)	18 (60)
Varicella zoster virus	14 (12.3)	2 (14.2)	7 (9.9)	5 (17.2)
Herpes Simplex virus 1	9 (7.9)	8 (57.1)	1 (1.4)	0 (0)
Herpes Simplex virus 2	2 (1.8)	0 (0)	1 (1.4)	1 (3.4)
Epstein-Barr-Virus	2 (1.8)	1 (7.1)	1 (1.4)	0 (0)
Influenza virus A/B	1 (0.9)	1 (7.1)	0 (0)	0 (0)
** Bacterial**	**34 (23)**	**1 (6.7)**	**18 (20.2)**	**15 (34.1)**
* Streptococcus pneumoniae*	16 (48.5)		9 (50)	7 (46.7)
* Neisseria meningitidis*	4 (12.1)		3 (16.7)	1 (6.7)
* Streptococcus ssp.*	4 (12.1)		2 (11.1)	2 (13.3)
* Haemophilus influenzae*	2 (6.1)		1 (5.6)	1 (6.7)
* Listeria monocytogenes*	1 (3)		0 (0)	1 (6.7)
* Staphylococcus aureus*	1 (3)		0 (0)	1 (6.7)
* Tuberculosis*	3 (9.1)		2 (11.1)	1 (6.7)
* Borrelia burgdorferi*	2 (6.1)		1 (5.6)	1 (6.7)
* Treponema pallidum*	1 (0.7)	1 (6.7)	0 (0)	0 (0)
*Immune-mediated cause*	13 (5)	13 (28.3)	0 (0)	0 (0)
NMDA-Receptor-Antibody	4 (30.7)	4 (30.7)		
LGI1 Antibody	4 (30.7)	4 (30.7)		
Caspr2	2 (15.4)	2 (15.4)		
Anti-Hu	1 (7.7)	1 (7.7)		
GAD	1 (7.7)	1 (7.7)		
SREAT	1 (7.7)	1 (7.7)		
*Other*	5 (1.9)	0 (0)	0 (0)	5 (5.9)
Intravenous Immunoglobuline	3 (60)			3 (60)
Craniopharyngioma	1 (20)			1 (20)
Autoimmune Disease	1 (20)			1 (20)
*Unknown cause*	92 (35.7)	18 (39.1)	38 (29.9)	35 (41.2)

*Legend*: Data are the number of patients with each clinically defined syndrome (%). Streptococcus ssp.: pyogenes, viridans, milleri, agalactiae; *NMDA* methyl D-aspartate receptor, *LGI-1* leucine-rich, glioma inactivated 1, *Caspr2* contact associated protein 2, *GAD* glutamic acid decarboxylase, *SREAT* steroid-responsive encephalopathy with autoimmune thyroiditis

TBEV was the most frequently detected cause of meningoencephalitis or meningitis (65/258 cases, 25%) in our overall patient population; 58 (89%) presented with meningoencephalitis (including 9 cases of meningoencephalomyelitis and meningoencephalomyeloradiculitis), 6 (9%) with meningitis and 1 (2%) with encephalitis. In the meningoencephalitis group, 6 patients were classified as having meningoencephalomyelitis (causes: 2 VZV and 4 TBE), and 7 were classified as having meningoencephalomyeloradiculitis (causes: 5 TBE, 1 unknown and 1 suspected parainfectious cause with *Bartonella henselae* infection). In the encephalitis group, in 13/46 (28%) patients, an autoimmune cause was found. Recurrent meningitis was seen in 4 patients: one had 2 episodes within 6 months and was later suspected to have systemic lupus erythaematodes, one had 3 episodes (2007 and 2 episodes in 2017) without known cause, one had 2 episodes of enteroviral meningitis within 3 years and one had 2 episodes of self-limiting treatment-induced meningitis within 3 months during treatment of myelodysplastic syndrome with antibiotics, virostatic and corticosteroid treatment.

### Clinical presentation

As shown in detail in Table 1, the median time from the onset of signs and symptoms until hospital admission was 5.5 days in encephalitis, 7 days in nonbacterial meningoencephalitis, 4 days in nonbacterial meningitis and 2 days in bacterial meningoencephalitis/meningitis.

Three patients presented with meningitis within 48 h after IVIG infusion with the main symptoms of headache, phono- and/or photophobia and nausea and had mild pleocytosis with normal protein values. All patients had a mRS of 0 at hospital discharge.

### Follow-up

The telephone follow-up interview took place at a median of 16 months (range 2-40 months) after hospital discharge (Supplementary Table 1) in 193 patients. Forty-six patients were lost to follow-up, and 19 died; 9 died due to acute encephalitis or meningoencephalitis. All interviewed patients were living at home, and the majority (96-100%) were able to cook their own meal, perform the laundry, and use public transport unaided independently of the diagnostic group. However, only 50% of encephalitis, 75% of bacterial meningoencephalitis/meningitis, 81% of nonbacterial meningoencephalitis and 94% of nonbacterial meningitis survivors were able to restart work or studies to the same extent as before the acute illness. Overall, 37% of patients indicated not feeling completely fit again (encephalitis 65%, nonbacterial meningoencephalitis 39%, nonbacterial meningitis 16%, bacterial meningoencephalitis/meningitis 53%). When asked whether to still feel more rapidly exhausted physically or mentally compared to before the acute illness, 47% of patients agreed (encephalitis 70%, nonbacterial meningoencephalitis 45%, nonbacterial meningitis 32%, bacterial meningoencephalitis/meningitis 71%).

Persisting neurological manifestations were reported by 56% of patients irrespective of elapsed time since acute illness (encephalitis 83%, nonbacterial meningoencephalitis 54%, nonbacterial meningitis 42%, bacterial meningoencephalitis/meningitis 71%, Supplementary Table 1). The most frequent manifestations were EDS and/or fatigue (34, 95% CI 27-41, Supplementary Fig. 1), cognitive impairment (22%; 29-16), headache (14%; 9-20) and epileptic seizures (11%; 6-16). Regarding headache, 9% (4-13) indicated suffering from headache less than 15 days per month, and 6% (2-9) reported suffering more than 15 days per month.

### Follow-up questionnaires

**Fig. 2:**
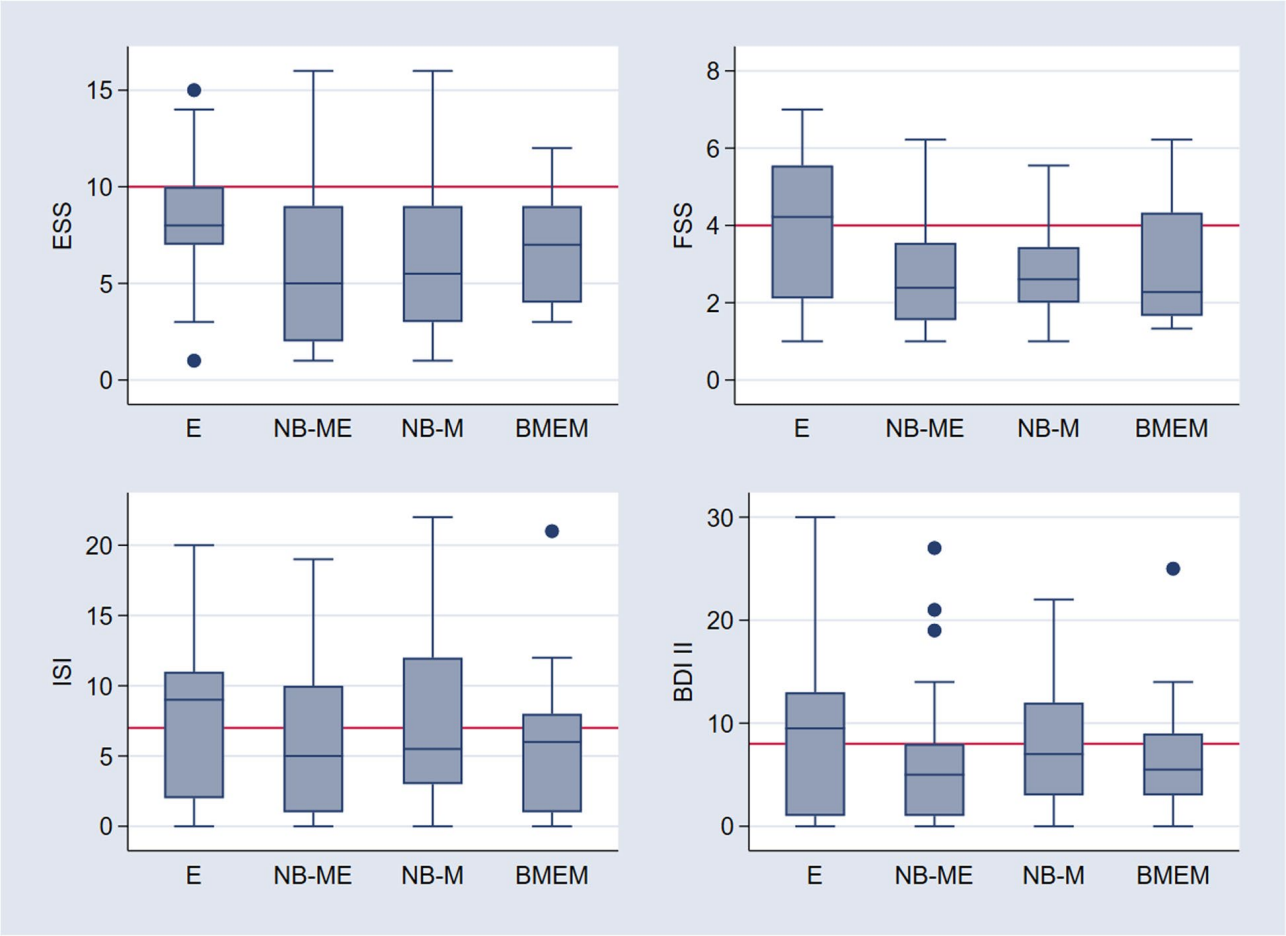
Comparison of scores of Epworth Sleepiness Score, Fatigue Severity Score, Insomnia Severity Index and Beck Depression Inventory II between encephalitis, non-bacterial meningoencephalitis, non-bacterial meningitis and bacterial meningoencephalitis or meningitis at follow up. Legend: *ESS* Epworth Sleepiness Score, *FSS* Fatigue Severity Scale, *ISI* Insomnia Severity Index, *BDI II* Beck Depression Inventory II; *E* Encephalitis, *NB-ME* Non-bacterial Meningoencephalitis, *NB-M* Non-bacterial Meningitis, *BMEM* Bacterial Meningoencephalitis/Meningitis, *boxes* medians and interquartile ranges, red line indicates cut off for pathological scores

Overall, 97 patients returned questionnaires (Fig. 2), including the Epworth Sleepiness Score (ESS), Fatigue Severity Score (FSS), Insomnia Severity Index (ISI) and Beck Depression Index II (BDI II). Overall, the proportions of pathological scores were 23% (95% CI 15-32) for ESS (cut-off > 10), 24% (16-33) for FSS (cut-off > 4), 40% (31-50) for ISI (cut-off > 7) and 31% (23-41) for BDI II (cut-off > 8). The proportion of pathological scores was not significantly different between groups (ESS *p* = 0.258, FSS *p =* 0.06 ISI *p* = 0.403, BDI II *p* = 0.077). Time since hospital discharge did not significantly influence any score value (ESS R = 0.0002, 95% CI − 0.86-0.1, *p* = 0.885; FSS R = 0, 95% CI -0.04-0.04, *p* = 0.967; ISI R = 0.012, 95% CI -0.206-0.061, *p* = 0.285; BDI II R = 0.0001, 95% CI -0.148-0.16, *p* = 0.08).

In a set of questions regarding sleep-wake disorders, 39 and 42% (95% CI 30-49 and 33-52, respectively) indicated suffering from excessive daytime sleepiness (agreed to questions: “I am tired during the day and I have to fight to stay awake and against sleeping in” and “It happens frequently, that I am forced to take a nap.”). However, 54% (95% CI 44-63) also indicated suffering from fatigue (agreed to question: “During the Day I feel exhausted and tired, however I am not able to sleep in when given a possibility to nap.”). New onset of daytime SWD, such as fatigue and EDS since suffering from the acute illness, was indicated by 25% (95% CI 17-34); 33% (24-43) thereof indicated having been suffering from the same problems already before the acute illness, and 42% (33-52) could not indicate clear onset of fatigue or EDS.

Overall, 22 patients out of 97 answered all 4 diagnostic RLS questions positively and accordingly fulfilled the diagnostic criteria of RLS. Twelve patients indicated having experienced new onset of RLS since the acute illness.

Five out of 97 patients indicated new onset of hypnagogic or hypnopompic hallucinations since the acute illness. No patient reported new onset of episodes indicating REM-sleep behaviour disorder, sleep paralysis or cataplexy since surviving the acute illness.

## Discussion

There were three main findings in this study. First, in our hospital in Switzerland, TBEV is the most important overall infectious cause of encephalitis, meningoencephalitis and meningitis, presenting as meningoencephalitis in the majority of patients. Second, only one-third of patients remain without a detectable cause of acute illness. Third, neurologic sequelae, most importantly cognitive impairment, fatigue and headache, were self-reported in a significant proportion not only of encephalitis, meningoencephalitis and bacterial meningitis survivors but also of nonbacterial meningitis patients up to 40 months after surviving the acute illness.

HSV was the most common cause of encephalitis, followed by immune-mediated causes. Therefore, together with VZV encephalitis, almost three-quarters of our encephalitis cases had a treatable cause. Depending on geographic location, these findings are in line with other European countries [1, 3, 33, 34]. While early detection and treatment of herpes encephalitis is essential for prevention of fatal outcome [35], recognition of autoimmune causes is of utmost importance since they belong to an expanding group of potentially treatable and curable causes of encephalitis and likely benefit from early immunosuppressive treatment regimens [36, 37]. Nonbacterial meningoencephalitis was most importantly caused by TBEV, since it is endemic in most parts of Switzerland with a nationwide estimated incidence of 6.83 cases per 100,000 unvaccinated inhabitants and nationwide vaccine coverage of only 42% for 1 dose and 33% for full vaccination with 3 doses in 2018 [38]. Since TBE vaccination is generally well tolerated with high rates of seroconversion, it has been recommended for all age groups above 1 year in highly endemic areas and for individuals at risk in areas with a lower incidence, including travellers with outdoor activities [22]. Higher vaccine coverage in Switzerland would most likely have an effect on local meningoencephalitis incidence; many cases could therefore be prevented.

In line with large European studies, *Streptococcus pneumoniae* was the main detected cause of bacterial meningitis/meningoencephalitis [2]. Finally, in line with other studies, including a large cohort study from the United Kingdom [1], enterovirus was the most important cause of nonbacterial meningitis. Interestingly, three patients were hospitalized with drug-induced meningitis due to intravenous immunoglobulin (IVIG) treatment, a rare side effect described in the literature occurring in up to 1% of cases receiving IVIG therapy [39]. Overall, 35% of cases remained without a known cause, which is in line with other studies [1, 3–5]. Causative agents in this group of patients remain speculative. However, recent studies have demonstrated increased diagnostic yield using next-generation sequencing methods to identify further causative infectious pathogens [40, 41].

At follow-up – independent of elapsed time of up to 40 months after acute illness – more than one-third of patients indicated that they had not regained full fitness, and more than half complained about neurological sequelae, despite functioning well in everyday activities such as cooking, doing laundry and financial affairs. However, neurological sequelae were frequently reported, mostly in encephalitis (83%) and bacterial meningoencephalitis/meningitis survivors (71%) but also in meningoencephalitis (54%) and nonbacterial meningitis (42%) survivors. Comparable findings have been described for encephalitis and meningoencephalitis, including TBE [10, 12, 13]. Our results are also in line with studies on viral meningitis, demonstrating long-lasting sequelae despite its ostensibly benign course [1, 42–44].

However, only half of encephalitis and 75% of bacterial meningoencephalitis/meningitis survivors were able to return to work. In a recent cohort study on community-acquired bacterial meningitis in adults, only 62% were able to return to work [45], and our higher proportion of survivors able to return to work might be due to a selection bias in our telephone interview, as discussed below.

To our knowledge, only a few studies have been published using standardized questionnaires on EDS, fatigue and insomnia in the field of encephalitis, meningoencephalitis and meningitis [17, 18]. In our study, EDS and/or fatigue were frequently reported in the follow-up; however, precise discrimination between the two clinically different symptoms was not possible. Insomnia/disturbed nighttime sleep was less frequently reported. Thirty-one percent of patients scored a pathological BDI II; however, the majority of patients had scores below 20 points, except encephalitis, with up to 30 points indicating mild to moderate depression. Since fatigue and insomnia may be key features of depression, interactions must be taken into account when using questionnaires in study settings as well as routine clinical practice. Symptoms need to be carefully evaluated to distinguish the origin of overlapping signs and symptoms.

The limitations of our study are the retrospective analysis of clinical cases in the acute phase with less precise data on clinical signs and symptoms and therefore diagnostic classification. In the retrospective use of patient data, we probably missed cases where general consent for further data use was unavailable. The telephone follow-up and the relatively low number of returned questionnaires might introduce bias in two possible directions.

First, it is possible that survivors with better outcomes might have been more willing to participate in the study. Since it is mostly not possible to contact severely disabled patients via telephone, this could potentially account for our number of lost-for follow-up patients. Therefore, it could be speculated that the true overall outcome of our full study population may be worse than that described in our study. Alternatively, it is possible that patients with sequelae may have been more motivated to participate in the telephone interview than those who had completely recovered. This scenario would therefore imply an overall better outcome than that reported in our study. Important value to the study and an attempt to solve these problems would have been the inclusion of a control group hospitalized for other acute illnesses treated in the hospital not affecting the CNS. Moreover, outcome data are difficult to interpret regarding the different time points when the follow-up survey took place. Taken together, our results on long-term outcomes have to be interpreted with care and need validation in further, ideally prospective studies. Important points for future studies are to include a personal follow-up visit for a detailed interview and a physical examination to better interpret and underline subjective complaints as well as evaluation of quality of life and the impact of persisting signs and symptoms on everyday life. Another important topic not addressed in our study is the importance of elapsed time from symptom onset until diagnosis and treatment or even time from “door to diagnosis and treatment” and its effect on functional long-term outcome.

Encephalitis and meningoencephalitis are often difficult to distinguish in routine clinical practice and have overlapping features and causes. This can also be seen in our encephalitis patients, whereof 26% had headache, and in 2 patients, even signs of meningism were documented. Therefore, clinical signs and symptoms may depict a continuum between meningoencephalitis and encephalitis for the diagnostic work-up at the emergency department. Nevertheless, it is important to try to classify the clinical syndrome to direct the diagnostic steps in the right direction of the most likely and most important causes.

## Conclusions

With this observational study including 258 patients, we demonstrate the importance of TBEV as the major cause of encephalitis, meningoencephalitis and meningitis cases in our geographic region and its primary clinical presentation as meningoencephalitis. Furthermore, we were able to assess long-lasting neurological sequelae not only after encephalitis, meningoencephalitis or bacterial meningitis but also after nonbacterial meningitis in a relevant proportion of patients up to 40 months post infection.

## Supplementary Information

**Additional file 1: Supplementary Table 1.** Follow up Interview. **Supplementary Fig. 1.** Follow-up Interview: self-reported neurological signs and symptoms persist up to 40 months after hospitalization. For each neurological symptom, individual timing of the follow-up interview relative to hospitalization is illustrated with dot plots. Individual data are summarized as Box plots.

## Data Availability

Anonymized data will be shared with qualified investigators upon contacting the corresponding author.

## Abbreviations

BDI-II: Beck Depression Inventory II
Caspr2: Contactin associated protein 2
CI: Confidence Interval
CMV: Cytomegalovirus
CNS: Central Nervous System
CSF: Cerebrospinal Fluid
EBV: Ebstein Barr Virus
EDS: Excessive Daytime Sleepiness
EEG: Electroencephalography
ESS: Epworth Sleepiness Scale
FSS: Fatigue Severity Scale
GAD: Glutamic acid decarboxylase
GCS: Glasgow Coma Scale
HSV: Herpes Simplex Virus
HHV: Human Herpes Virus
ISS: Insomnia Severity Index
IQR: Interquartile range
LGI-1: Leucine-rich, glioma inactivated 1
mRS: Modified Rankin Score
NMDA: Methyl D-aspartate receptor
PCR: Polymerase Chain Reaction
REDCap: Research Electronic Data Capture
RLS: Restless Legs Syndrome
SREAT: Steroid-responsive encephalopathy with autoimmune thyroiditis
SWD: Sleep Wake Disorders
TBE: Tick borne encephalitis
TBEV: Tick borne encephalitis virus
VZV: Varicella Zoster Virus
WNV: West Nile Virus

## Declarations

### Competing of interests

On behalf of all authors, the corresponding author states that there are no competing of interests.
