# Supplementary material for: Meningitis, meningoencephalitis and encephalitis in Bern: an observational study of 258 patients
Source: BMC Neurol. 2021 Dec 6;21:474. doi: 10.1186/s12883-021-02502-3 (PMC8647376; doi:10.1186/s12883-021-02502-3)
Supplement: Supplementary file 1 — Additional file 1: Supplementary Table 1. Follow up Interview. Supplementary Fig. 1. Follow-up Interview: self-reported neurological signs and symptoms persist up to 40 months after hospitalization. For each neurological symptom, individual timing of the follow-up interview relative to hospitalization is illustrated with dot plots. Individual data are summarized as Box plots. [file 12883_2021_2502_MOESM1_ESM.zip › 12883_2021_2502_MOESM1_ESM.docx]

Supplementary table 1 Follow up Interview

|  | All | Encephalitis | Non-bacterial Meningo-encephalitis | Non-bacterial Meningitis | Bacterial Meningo-encephalitis or Meningitis |
| --- | --- | --- | --- | --- | --- |
| n | 162 | 23 | 71 | 50 | 18 |
| Median months since Hospital discharge months (IQR) | 16 (15) | 14 (12) | 18 (15) | 14 (13) | 25 (13) |
| Living at home* | 100 | 100 | 100 | 100 | 100 |
| Able to restart work to same extent | 80 (73-86) | 50 (30-70) | 81 (70-89) | 94 (82-98) | 75 (49-90) |
| Able to manage own financial affairs | 96 (91-98) | 86 (65-96) | 97 (89-99) | 98 (87-100) | 94 (66-99) |
| Able to cook own meal | 97 (93-99) | 96 (74-99) | 97 (89-99) | 98 (86-100) | 100 |
| Able to do own laundry | 97 (92-99) | 96 (74-99) | 96 (87-99) | 98 (86-100) | 100 |
| Able to use public transport unaided | 96 (92-98) | 91 (70-98) | 99 (90-100) | 96 (85-99) | 94 (66-99) |
| Persisting neurological signs and symptoms* | 56 (48-63) | 83 (62-93) | 54 (42-65) | 42 (29-56) | 71 (46-87) |
| Feeling not completely fit again | 37 (30-45) | 65 (44-86) | 39 (29-51) | 16 (8-29) | 53 (30-75) |
| Feeling more rapidly exhausted physically or mentally | 47 (40-55) | 70 (48-85) | 45 (34-57) | 32 (20-46) | 71 (46-87) |
| New onset of excessive daytime sleepiness (EDS) and/or fatigue | 34 (27-41) | 57 (30-75) | 34 (24-46) | 16 (8-29) | 53 (30-75) |
| New onset insomnia | 15 (10-21) | 13 (4-34) | 13 (7-23) | 14 (7-27) | 29 (13-54) |

Table legend: *Data are % (95% CI)

Supplementary figure 1 Follow-up Interview: self-reported neurological signs and symptoms persist up to 40 months after hospitalization. For each neurological symptom, individual timing of the follow-up interview relative to hospitalization is illustrated with dot plots. Individual data are summarized as Box plots.
