# Supplementary material for: Meningitis, meningoencephalitis and encephalitis in Bern: an observational study of 258 patients
Source: BMC Neurol. 2021 Dec 6;21:474. doi: 10.1186/s12883-021-02502-3 (PMC8647376; doi:10.1186/s12883-021-02502-3)
Supplement: Supplementary file 1 — Additional file 1: Supplementary Table 1. Follow up Interview. Supplementary Fig. 1. Follow-up Interview: self-reported neurological signs and symptoms persist up to 40 months after hospitalization. For each neurological symptom, individual timing of the follow-up interview relative to hospitalization is illustrated with dot plots. Individual data are summarized as Box plots. [file 12883_2021_2502_MOESM1_ESM.zip › 12883_2021_2502_MOESM3_ESM.pdf]

Sehr geehrter Patientin, sehr geehrter Patient!

Sie wurden vor kurzem telefonisch über die Teilnahme an unserer Umfrage (Studie) zu Folgen nach Hirnentzündungen informiert und haben eingewilligt an unserer Umfrage teilzunehmen. Wir möchten Sie bitten, nun folgende Fragebögen auszufüllen und an uns im beigelegten und bereits frankierten Umschlag zu retournieren.

Sollten Sie Fragen haben, können Sie sich jederzeit an das Studienteam wenden.

Dr. Anelia Dietmann, Oberärztin Neurologie, Telefon: 031 632 60 48
anelia.dietmann@insel.ch

Anamaria Ungureanu, Assistenzärztin Neurologie, Telefon 031 632 21 11
anamaria.ungureanu@insel.ch

Vielen Dank, dass Sie sich die Zeit für die Teilnahme an unserer Umfrage nehmen!

Nachname…………………………………………………………………………….

Vorname……………………………………………………………………………….

Geburtsdatum………………………………………………………………………

Heutiges Datum…………………………………………………………………….

Bitte nicht ausfüllen!

PatientenID: EME……………………..

Datum…………………………………………..

Epworth Sleepiness Scale		
Die folgende Frage bezieht sich auf Ihr normales Alltagsleben in den letzten 4 Wochen:<br><br>**Für wie wahrscheinlich halten Sie es, dass Sie in einer der folgenden Situationen einnicken oder einschlafen würden? Nicht gemeint ist damit, dass Sie sich nur müde fühlen.**<br><br>Wenn Sie gewisse Situationen in der letzten Zeit nicht erlebt haben, versuchen Sie bitte, sich vorzustellen, wie es Ihnen dabei ergangen wäre.<br><br>Wählen Sie aus der folgenden Skala die für die entsprechende Frage am besten zutreffende Zahl.<br>0 = würde niemals einschlafen oder einnicken<br>1 = würde kaum einschlafen oder einnicken<br>2 = würde möglicherweise einschlafen oder einnicken<br>3 = würde sehr wahrscheinlich einschlafen oder einnicken		
**Aktivität**		**Wert**
Im Sitzen Lesen		
Beim Fernsehen		
Wenn Sie passiv (als Zuhörer) in der Öffentlichkeit sitzen (zB im Theater, Kino, Vortrag, Sitzung)		
Als Beifahrer im Auto während einer einstündigen Fahrt ohne Pause		
Wenn Sie sich am Nachmittag hingelegt haben um auszuruhen		
Wenn Sie sitzen und sich mit jemandem unterhalten		
Wenn Sie nach dem Mittagessen (ohne Alkohol) ruhig sitzen		
Wenn Sie als Fahrer eines Autos verkehrsbedingt einige Minuten halten müssen (zB rote Ampel, Stau)		

Fragebogen zu Müdigkeit und Erschöpfung Fatigue Severity Scale							
Ich finde, dass während der vergangen Woche folgendes zutraf:							
	Stimme gar nicht zu						Stimme vollkommen zu
Ich bin weniger motiviert, wenn ich müde bin.	1	2	3	4	5	6	7
Körperliche Bewegung macht mich müde.	1	2	3	4	5	6	7
Ich ermüde rasch.	1	2	3	4	5	6	7
Meine Müdigkeit beeinträchtigt meine körperliche Leistungsfähigkeit.	1	2	3	4	5	6	7
Meine Müdigkeit bereitet mir oft Probleme.	1	2	3	4	5	6	7
Meine Müdigkeit verhindert längerdauernde körperliche Tätigkeiten.	1	2	3	4	5	6	7
Meine Müdigkeit beeinträchtig mich, gewisse Pflichten und Verantwortungen zu erfüllen.	1	2	3	4	5	6	7
Meine Müdigkeit gehört zu den drei Beschwerden, die mich am meisten behindern.	1	2	3	4	5	6	7
Meine Müdigkeit beeinträchtigt meine Arbeit, meine Familie oder mein soziales Leben.	1	2	3	4	5	6	7

Beck-Depressions- Inventar (BDI)

Dieser Fragebogen enthält 21 Gruppen von Aussagen. Bitte lesen Sie jede Gruppe sorgfältig durch. Suchen Sie dann die eine Aussage in jeder Gruppe heraus, die am besten beschreibt, wie Sie sich in dieser Woche einschließlich heute gefühlt haben und kreuzen Sie die dazugehörige Ziffer (0, 1 2 oder 3) an. Falls mehrere Aussagen einer Gruppe gleichermaßen zutreffen, können Sie auch mehrere Ziffern markieren, Lesen Sie auf jeden Fall alle Aussagen in jeder Gruppe, bevor Sie Ihre Wahl treffen.

1 ⓪ Ich bin nicht traurig.
① Ich bin traurig.
② Ich bin die ganze Zeit traurig und komme nicht davon los.
③ Ich bin so traurig oder unglücklich, dass ich es kaum noch ertrage,

2 ⓪ Ich sehe nicht besonders mutlos in die Zukunft,
① Ich sehe mutlos in die Zukunft.
② Ich habe nichts, worauf ich mich freuen kann.
③ Ich habe das Gefühl, dass die Zukunft hoffnungslos ist und das die Situation nicht besser werden kann.

3 ⓪ Ich fühle mich nicht als Versager.
① Ich habe das Gefühl, öfter versagt zu haben als der Durchschnitt.
② Wenn ich auf mein Leben zurückblicke, sehe ich bloß eine Menge Fehlschläge.
③ Ich habe das Gefühl, als Mensch ein völliger Versager zu sein.

4 ⓪ Ich kann die Dinge genauso genießen wie früher,
① Ich kann die Dinge nicht mehr so genießen wie früher.
② Ich kann aus nichts mehr eine echte Befriedigung ziehen.
③ Ich bin mit allem unzufrieden oder gelangweilt,

5 ⓪ Ich habe keine Schuldgefühle,
① Ich habe häufig Schuldgefühle
② Ich habe fast Immer Schuldgefühle.
③ Ich habe immer Schuldgefühle,

6 ⓪ Ich habe nicht das Gefühl, gestraft zu sein.
① Ich habe das Gefühl vielleicht bestraft zu werden.
② Ich erwarte, bestraft zu werden,
③ Ich habe das Gefühl bestraft zu gehören,

7 ⓪ Ich bin nicht von mir enttäuscht.
① Ich bin von mir enttäuscht.
② Ich finde mich fürchterlich.
③ Ich hasse mich,

8 ⓪ Ich habe nicht das Gefühl schlechter zu sein als die anderen.
① Ich kritisiere mich wegen meiner Fehler und Schwächen.
② Ich mache mir die ganze Zeit Vorwürfe wegen meiner Mängel.
③ Ich gebe mir für alles die Schuld, was schiefgeht.

9 ⓪ Ich denke nicht daran, mir etwas anzutun.
① Ich denke manchmal an Selbstmord, aber ich würde es nicht tun.

② Ich möchte mich am liebsten umbringen.
③ Ich würde mich umbringen, wenn ich es könnte.

10 ⓪ Ich weine nicht öfter als früher,
① Ich weine jetzt mehr als früher,
② Ich weine jetzt die ganze Zeit.
③ Früher konnte ich weinen, aber jetzt kann ich es nicht mehr, obwohl ich es möchte.

11 ⓪ Ich bin nicht reizbarer als sonst
① Ich bin jetzt leichter verärgert oder gereizt als früher,
② Ich fühle mich dauernd gereizt.
③ Die Dinge, die mich früher geärgert haben, berühren mich nicht mehr.

12 ⓪ Ich habe nicht das Interesse an Menschen verloren,
① Ich interessiere mich jetzt weniger für Menschen als früher.
② Ich habe mein Interesse an anderen Menschen zum größten Teil verloren.
③ Ich habe mein ganzes Interesse an anderen Menschen verloren,

13 ⓪ Ich bin so entschlussfreudig wie immer.
① Ich schiebe Erledigungen jetzt öfter als früher auf,
② Es fällt mir jetzt schwerer als früher, Entscheidungen zu treffen.
③ Ich kann überhaupt keine Entscheidungen mehr treffen.

14 ⓪ Ich habe nicht das Gefühl, schlechter auszusehen als früher.
① Ich mache mir Sorgen, daß ich alt oder unattraktiv aussehe.
② Ich habe das Gefühl, daß in meinem Aussehen Veränderungen eintreten.
③ Ich finde mich hässlich.

15 ⓪ Ich kann so gut arbeiten wie früher.
① Ich muss mir einen Ruck geben, bevor ich eine Tätigkeit in Angriff nehme.
② Ich muss mich zu jeder Tätigkeit zwingen.
③ Ich bin unfähig zu arbeiten.

16 ⓪ Ich schlafe so gut wie sonst,
① Ich schlafe nicht mehr so gut wie früher.
② Ich wache 1 bis 2 Stunden früher auf als sonst, und es fällt mir schwer, wieder einzuschlafen.
③ Ich wache mehrere Stunden früher auf als sonst und kann nicht mehr einschlafen.

17 ⓪ Ich ermüde nicht stärker als sonst
① Ich ermüde schneller als früher.
② Fast alles ermüdet mich,
③ Ich bin zu müde, um etwas zu tun

18 ⓪ Mein Appetit ist nicht schlechter als sonst.
① Mein Appetit ist nicht mehr so gut wie früher.
② Mein Appetit hat sehr stark nachgelassen.
③ Ich habe überhaupt keinen Appetit mehr.

19 ⓪ Ich habe in letzter Zeit kaum abgenommen.
① Ich hebe mehr als 2 Kilo abgenommen.
② ich habe mehr als 5 Kilo abgenommen.

③ Ich habe mehr als 8 Kilo abgenommen,
Ich esse absichtlich weniger, um abzunehmen: [ ]ja [ ] Nein

20 ⓪ Ich mache mir keine größeren Sorgen um meine Gesundheit als sonst.
① Ich mache mir Sorgen über körperliche Probleme, wie Schmerzen, Magenbeschwerden oder Verstopfung,
② Ich mache mir so große Sorgen über gesundheitliche Probleme, dass es mir schwerfällt, an Etwas anderes zu denken.
③ Ich mache mir so große Sorgen über gesundheitliche Probleme, dass ich an nichts anderes Mehr denken kann.

21 ⓪ Ich habe in letzter Zeit keine Veränderung meines Interesses an Sex bemerkt.
① Ich interessiere mich weniger für Sex als früher.
② Ich interessiere mich jetzt viel weniger für Sex.
③ Ich habe das Interesse an Sex völlig verloren.

Insomnie-Schweregrad-Index (ISI)

1. Bitte stufen Sie den aktuellen (d.h. **innerhalb der letzten zwei Wochen**) Schweregrad Ihrer Schlafprobleme ein.

gar nicht = 15-30 min<br>mild = 30-45 min<br>mittelmäßig = 45-90 min<br>schwer = 90-120 min<br>sehr schwer > 120 min	*gar nicht*	*wenig*	*mittelmäßig*	*schwer*	*sehr schwer*
1.a Schwierigkeiten, einzuschlafen	0	1	2	3	4
1.b Schwierigkeiten, durchzuschlafen	0	1	2	3	4
1.c Problem, zu früh aufzuwachen	0	1	2	3	4

2. Wie zufrieden waren Sie **innerhalb der letzten zwei Wochen** mit Ihrem aktuellen Schlafmuster?

sehr zufrieden				sehr unzufrieden
0	1	2	3	4

3. In welchem Ausmass hat die Schlafproblematik Ihre tägliche Funktionsfähigkeit (z.B. Tagesmüdigkeit, Fähigkeit bei der Arbeit/ Hausarbeit zu funktionieren, Konzentration, Gedächtnis, Stimmung etc**.)** **innerhalb der letzten zwei Wochen** beeinträchtigt?

überhaupt keine Beeinträchtigung	ein wenig	einigermassen	grosse	sehr grosse Beeinträchtigung
0	1	2	3	4

4. Wie erkennbar ist Ihre Schlafproblematik bezüglich der Beeinträchtigung Ihrer Lebens-qualität Ihrer Meinung nach für andere?

gar nicht erkennbar	ein wenig	einigermassen	deutlich	sehr deutlich erkennbar
0	1	2	3	4

5. Wie besorgt oder bekümmert sind Sie wegen Ihrer aktuellen Schlafproblematik?

gar nicht	ein wenig	einigermassen	sehr	sehr deutlich
0	1	2	3	4

Schlaffragebogen

Allgemeines zum Schlaf

1. Ich bin zufrieden mit meinem Schlaf ☐ Stimmt! ☐ mal so/mal so ☐ Stimmt nicht
2. Ich schlafe nachts zu viel ☐ Stimmt! ☐ mal so/mal so ☐ Stimmt nicht
3. Ich schlafe nachts zu wenig ☐ Stimmt! ☐ mal so/mal so ☐ Stimmt nicht
4. Ich habe einen tiefen und festen Schlaf ☐ Stimmt! ☐ mal so/mal so ☐ Stimmt nicht
5. Ich habe einen flachen, leichten Schlaf ☐ Stimmt! ☐ mal so/mal so ☐ Stimmt nicht
6. Ich empfinde meinen Schlaf als erholsam ☐ Stimmt! ☐ mal so/mal so ☐ Stimmt nicht
7. Ich habe Probleme nachts zu schlafen ☐ Stimmt! ☐ mal so/mal so ☐ Stimmt nicht

   Welche?

   ____

   ____

   ____

   ____

   ____

   ____

8. Bestanden die Schlafprobleme bereits vor Ihrer Hirnentzündung?

   ☐ Nein, seit damals neu aufgetreten

   ☐ Ja, schon vorher bestehend

   ☐ Weiss nicht

9. Wenn Ja in Frage 8, haben sich die Schlafprobleme durch die Hirnentzündung verschlimmert?

   ☐ Nein, gleich geblieben wie vorher

   ☐ Ja, verschlechtert seither

   ☐ Weiss nicht

Einschlafen

10. Um welche Zeit gehen Sie in der Regel zu Bett?

    werktags ______________________ Uhr

    am Wochenende oder im Urlaub ______________________ Uhr

11. Ich habe ein Problem einzuschlafen

    ☐ Stimmt, fast jeden Abend

    ☐ Stimmt,. 1-2x/Woche

    ☐ Stimmt, 2-3x/Monat

    ☐ Stimmt, aber nur ganz selten

    ☐ Nein, ich habe kein Problem einzuschlafen

12. Wie lange brauchen Sie in der Regel um einzuschlafen, nachdem Sie im Bett das Licht gelöscht haben? ______________________ Minuten

Durchschlafen

13. Ich habe ein Problem durchzuschlafen

    ☐ Stimmt, fast jede Nacht

    ☐ Stimmt,. 1-2x/Woche

    ☐ Stimmt, 2-3x/Monat

    ☐ Stimmt, aber nur ganz selten

    ☐ Nein, ich habe kein Problem durchzuschlafen

14. Wie viele Stunden schlafen Sie ca. pro Nacht? __________________Stunden

15. Ich schnarche nachts laut und störend

    ☐ Stimmt, fast jede Nacht

    ☐ Stimmt, gelegentlich

    ☐ Nein, stimmt nicht

    ☐ Weiss nicht, schlafe alleine

16. Ich wache oft nachts erschreckt auf und ringe um Luft oder hab ein beklemmendes Gefühl auf der Brust

    ☐ Stimmt, fast jede Nacht

    ☐ Stimmt, gelegentlich

    ☐ Nein, stimmt nicht

17. Ich schnarche seit der Hirnhautentzündung häufiger/lauter/störender

☐ Stimmt, fast jeden Morgen

☐ Stimmt nicht

☐ Weiss nicht/schlafe alleine

Aufwachen

18. Um welche Zeit erwachen Sie? (Format 07:00)

werktags ______________________

am Wochenende oder im Urlaub ________________________

19. Wie wachen Sie am Morgen auf?

☐ Selbst ☐ Wecker ☐ muss von anderer Person geweckt werden

20. Am Morgen erwache ich oft früher als gewollt

☐ Stimmt, fast jeden Morgen

☐ Stimmt, aber nur selten

☐ Stimmt nicht

☐ Weiss nicht

21. Am Morgen fühle ich mich häufig schlecht ausgeschlafen/unausgeschlafen

☐ Stimmt, fast jeden Morgen

☐ Stimmt, aber nur selten

☐ Stimmt nicht

☐ Weiss nicht

22. Am Morgen werde ich häufig nur langsam und unvollständig wach, ich muss häufig vergeblich versuchen aufzuwachen (zB mit mehreren Wecker)

☐ Stimmt, fast jeden Morgen

☐ Stimmt, aber nur selten

☐ Stimmt nicht

☐ Weiss nicht

23. Am Morgen fühle ich mich oft verwirrt, wie betrunken

    ☐ Stimmt, fast jeden Tag

    ☐ Stimmt, aber nur selten

    ☐ Stimmt nicht

    ☐ Weiss nicht

24. Wie viele Minuten brauchen Sie morgens, um in Gang zu kommen bzw. um sich richtig frisch zu fühlen? _______________Minuten

Tagsüber

25. Ich bin tagsüber müde und muss mich anstrengen, wach zu bleiben, ich muss oft gegen das Einnicken/Einschlafen ankämpfen

    ☐ Stimmt, fast jeden Tag

    ☐ Stimmt, aber nur selten

    ☐ Stimmt nicht

    ☐ Weiss nicht

26. Es passiert mir häufig, dass ich tagsüber einschlafe oder ein kurzes Schläfchen halten muss

    ☐ Stimmt, fast jeden Tag

    ☐ Stimmt, aber nur selten

    ☐ Stimmt nicht

    ☐ Weiss nicht

27. Ich fühle mich tagsüber oft müde, erschöpft und/oder energielos, ich schlafe dabei aber nicht ein

    ☐ Stimmt, fast jeden Tag

    ☐ Stimmt, aber nur selten

    ☐ Stimmt nicht

    ☐ Weiss nicht

28. Auf Grund meiner Müdigkeit/Schläfrigkeit habe ich Probleme in der Schulde/bei der Arbeit

    ☐ Stimmt, fast jeden Tag

    ☐ Stimmt, aber nur selten

    ☐ Stimmt nicht

    ☐ Weiss nicht

29. Wie oft machen Sie einen Mittagsschlaf/Nickerchen?

☐ Nie

☐ würde gerne, kann aber nicht einschlafen

☐ würde gerne, habe aber keine Zeit

☐ 1-2x/Wo

☐ fast täglich oder täglich

Sind diese Nickerchen erholsam? ☐ Nein ☐ Ja ☐ weiss nicht

30. Bestanden die Beschwerden von Frage 25-29 bereits vor Ihrer Hirnentzündung?

☐ Nein, sie sind seither neu aufgetreten seither

☐ Ja, das hatte ich schon vorher

☐ Weiss nicht

31. Wenn Ja, haben sich die Probleme wach zu bleiben durch die Hirnentzündung verschlimmert?

☐ Nein, sie sind gleich geblieben wie vor der Erkrankung

☐ Ja, es ist schlechter geworden seither

☐ Weiss nicht

Sonstiges

32. Haben Sie als Kind schlafgewandelt?

☐ Nein ☐ Ja ☐ Weiss nicht

33. Haben Sie seit Ihrer Erkrankung schlafgewandelt?

☐ Nein ☐ Ja ☐ Weiss nicht

34. Sprechen Sie im Schlaf?

☐ Nein ☐ Ja, sehr selten ☐ Ja, manchmal ☐ Ja, häufig ☐ weiss nicht oder schlafe alleine

35. Kommt es vor, dass Sie im Schlaf schreien, schimpfen oder gewalttätig werden (als ob Sie Albträume ausleben würden)

☐ Nein ☐ Ja, sehr selten ☐ Ja, manchmal ☐ Ja, häufig ☐ weiss nicht oder schlafe alleine

36. Wenn Ja, ist das neu aufgetreten seit der Hirnentzündung?

☐ Nein, schon vorher bestehend ☐ Ja, neu aufgetreten ☐ Weiss nicht

37. Leiden Sie an unangenehmen Missempfindungen der Beine (oder Arme) mit Kribbeln, Ziehen, Jucken, Brennen, krampfartig oder Schmerzen?

☐ Nein ☐ Ja ☐ weiss nicht

Wenn Sie bei Frage 37. Nein angekreuzt haben, dann weiter zu Frage 38, anderenfalls:

Wenn Ja bei Frage 37, zwingt Sie das unangenehme Gefühl Ihre Glieder zu bewegen, zu dehnen, zu massieren, duschen, reiben oder herumzulaufen?

☐ Nein ☐ Ja ☐ weiss nicht

Wenn Ja bei Frage 37, verstärken sich die Beschwerden in Ruhe und verschwinden (oder werden deutlich besser) durch Bewegung und Aktivität?

☐ Nein ☐ Ja ☐ weiss nicht

Wenn Ja bei Frage 37, treten die Beschwerden vor allem abends und nachts auf und sind deutlich besser am Morgen?

☐ Nein ☐ Ja ☐ weiss nicht

Wenn Ja in Frage 37, sind die Beschwerden neu aufgetreten seit der Hirnentzündung?

☐ Nein, schon vorher bestehend ☐ Ja, neu aufgetreten ☐ Weiss nicht

38. Ich fühle mich oft kurz wie gelähmt, ich bin wach, kann mich aber nicht bewegen

- beim Einschlafen

☐ stimmt, fast jeden Abend ☐ stimmt, aber nur selten ☐ stimmt nicht ☐ weiss nicht

- im Schlaf

☐ stimmt, fast jeden Abend ☐ stimmt, aber nur selten ☐ stimmt nicht ☐ weiss nicht

- beim Aufwachen

☐ stimmt, fast jeden Abend ☐ stimmt, aber nur selten ☐ stimmt nicht ☐ weiss nicht

Wenn Sie dieses Gefühl kennen, ist es neu Aufgetreten seit der Hirnentzündung?

☐ Nein, schon vorher bestehend ☐ Ja, neu aufgetreten ☐ Weiss nicht

39. Oft habe ich während des Einschlafens den Eindruck als ob jemand im meinem Zimmer wäre, mich berühre oder in mein Zimmer eindringen wolle (traumähnliche Bilder noch im Wachzustand)

☐ stimmt, fast jeden Abend ☐ stimmt, aber nur selten ☐ stimmt nicht ☐ weiss nicht

Wenn Sie dieses Gefühl kennen, ist es neu Aufgetreten seit der Hirnentzündung?

☐ Nein, schon vorher bestehend ☐ Ja, neu aufgetreten ☐ Weiss nicht

40. Haben Sie jemals eine der folgenden Beschwerden bei Emotionen wie Lachen, Freude, Trauer oder Wut empfunden?

☐ Weiche Knie/Knieschlottern

☐ Absinken des Unterkiefers

☐ Vorfallen des Kopfes

☐ Sturz

☐ Kraftverlust der Muskeln

☐ Nein, keines der oben angeführten Symptome kenne ich

41. Wenn Sie diese Beschwerden kennen, sind sie neu aufgetreten seit Ihrer Hirnentzündung?

☐ Nein, schon vorher bestehend ☐ Ja, neu aufgetreten ☐ Weiss nicht

42. Wie oft träumen Sie bzw. wie oft können Sie sich an Träume erinnern oder wissen, dass Sie geträumt haben?

☐ nie ☐ einzelne Nächte ☐ 1x/Woche ☐ mehrmals pro Woche ☐ fast jede Nacht

43. Haben Sich seit Ihrer Hirnhautentzündung Träume geändert?

☐ ja, ich träume häufiger/kann mich häufiger an Träume erinnern

☐ ja, ich träume seltener/kann mich seltener an Träume erinnern

☐ nein, meine Träume sind so wie vor der Erkrankung

☐ weiss nicht

Vielen Dank für Ihre Zeit und Mitarbeit!!
