# Supplementary figures and images for: Meningitis, meningoencephalitis and encephalitis in Bern: an observational study of 258 patients
Source: BMC Neurol. 2021 Dec 6;21:474. doi: 10.1186/s12883-021-02502-3 (PMC8647376; doi:10.1186/s12883-021-02502-3)

# Neurologic signs and symptoms

Neurologic signs and symptoms

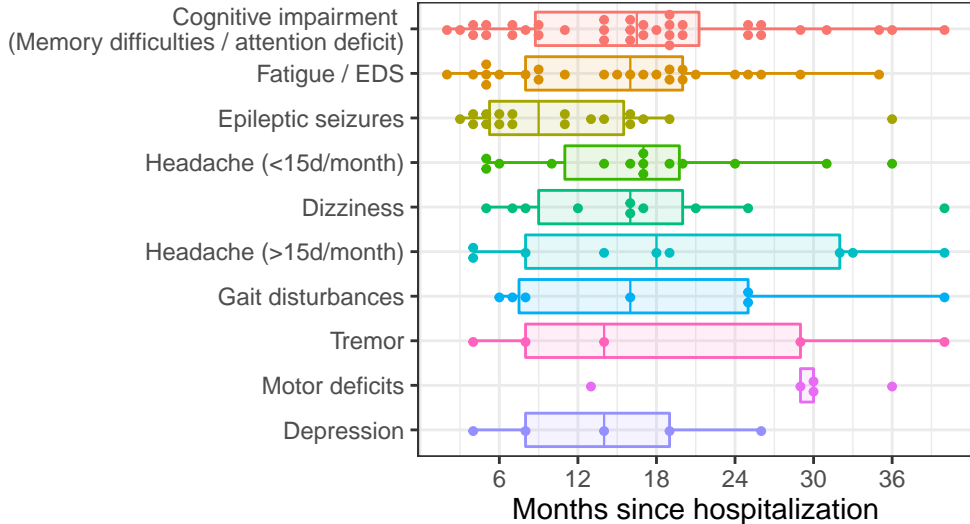

Supplement: Supplementary file 1 — Additional file 1: Supplementary Table 1. Follow up Interview. Supplementary Fig. 1. Follow-up Interview: self-reported neurological signs and symptoms persist up to 40 months after hospitalization. For each neurological symptom, individual timing of the follow-up interview relative to hospitalization is illustrated with dot plots. Individual data are summarized as Box plots. [file 12883_2021_2502_MOESM1_ESM.zip › 12883_2021_2502_MOESM2_ESM.pdf]
